# Modelling the evolution of novelty: a review

**DOI:** 10.1042/EBC20220069

**Published:** 2022-12-08

**Authors:** Enrico Sandro Colizzi, Paulien Hogeweg, Renske M.A. Vroomans

**Affiliations:** 1 Sainsbury Laboratory, University of Cambridge, 47 Bateman Street, CB2 1LR, Cambridge, U.K.; 2 Theoretical Biology and Bioinformatics, Universiteit Utrecht, Padualaan 8, 3584 CH, Utrecht, Netherlands

**Keywords:** evo-devo, evolution of novelty, multi-level evolution, multi-scale modelling

## Abstract

Evolution has been an inventive process since its inception, about 4 billion years ago. It has generated an astounding diversity of novel mechanisms and structures for adaptation to the environment, for competition and cooperation, and for organisation of the internal and external dynamics of the organism. How does this novelty come about? Evolution builds with the tools available, and on top of what it has already built – therefore, much novelty consists in repurposing old functions in a different context. In the process, the tools themselves evolve, allowing yet more novelty to arise.

Despite evolutionary novelty being the most striking observable of evolution, it is not accounted for in classical evolutionary theory. Nevertheless, mathematical and computational models that illustrate mechanisms of evolutionary innovation have been developed. In the present review, we present and compare several examples of computational evo–devo models that capture two aspects of novelty: ‘between-level novelty’ and ‘constructive novelty.’ Novelty can evolve between predefined levels of organisation to dynamically transcode biological information across these levels – as occurs during development. Constructive novelty instead generates a level of biological organisation by exploiting the lower level as an informational scaffold to open a new space of possibilities – an example being the evolution of multicellularity. We propose that the field of computational evo–devo is well-poised to reveal many more exciting mechanisms for the evolution of novelty. A broader theory of evolutionary novelty may well be attainable in the near future.

## Introduction

The emergence of novel features is the most charismatic consequence of evolution, pushing life from simple collections of molecules to the massive structures of trees and elephants we see today. Bioinformatic and experimental work has characterised much evolutionary novelty, in terms of its history and of its constituting components. Examples of macroscopic novel features are the major transitions [1], the co-option of feathers for flight from an initially thermoregulative, sensory, or display function [2–4], and the optical properties of metabolic enzymes in the eyes [5,6]. Several definitions of novelty exist that attempt to unify these and other motivating examples, which cover different aspects of novelty (reviewed in [7]). These definitions differ in the extent to which a feature must change or in the extent of its ecological or functional effect, before it counts as novel [8]. However, we lack a broad mechanistic theory for how novelty emerges through evolutionary processes. To begin constructing this theory, in the present review, we present several examples of computational models that show some features of evolutionary novelty. In doing so, we bypass the problem of defining evolutionary novelty. Therefore, we do not propose a new definition and we do not restrict ourselves to a single existing one. We believe that once we have captured evolutionary novelty in mathematical and computational models, we will then be in a better position to identify and organise novelty as has occurred in natural systems through evolutionary history.

There is, however, an apparent paradox in constructing evolutionary models to study novelty: if the kind of novelty that is the object of study is incorporated in the model, then the model cannot be said to evolve novelty – and therefore the model does not capture what it aims to explain. This is the case for models that predetermine the fitness values of traits (or combinations and trade-offs thereof), the social role of individuals in a population, or that produce gradual changes through an arms race.

In the following, we show results from models that escape this methodological paradox in two different ways. The starting point for our discussion is models of development and its evolution.

Models of development can show how a phenotype arises from the interactions between cells, intercellular signals and the environment. This approach has been used to study animal embryogenesis [9–11], plant morphogenesis and patterning [12–14], and slime mould development [15–18]. These models often explicitly incorporate processes occurring at multiple spatiotemporal scales, from the regulation of gene expression and/or dynamics of molecules inside cells, to cell movement and communication, to the shaping of whole tissues. The building blocks in these models structure the developmental process, which then results in an emergent phenotype. In other words, the level at which the model is specified is mapped by the developmental process to the level at which the outcome is observed; this constitutes a genotype-to-phenotype (GP) map [19–21]. Models of developmental evolution expand developmental models by making the genetic information (a genome, GRN or other developmental parameters) evolvable by mutation [22]. The phenotype resulting from the emerging developmental process may provide a competitive advantage to the individual possessing the genome, and lead to its selection within a population of other developing individuals. Because the structure of the GP map itself is not under explicit selection, nonlethal mutations can accumulate and cause qualitative changes in the developmental process. These qualitative changes are not predetermined by the modeller and are not explicitly included in the fitness function, and they are thus novel. Moreover, long-term evolution can shape the effect of mutations [23–26] – altering the potential for novelty in the process. This view on novelty resonates with a recent perspective that defines novelty as the effect of evolution across multiple scales [7].

### Between-level novelty

**Figure 1:**
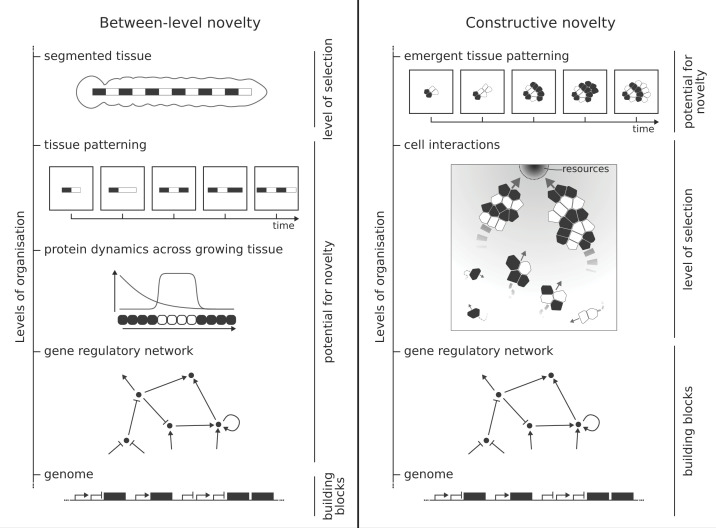
Two forms of evolutionary novelty in computational models **Between-level novelty**: Evolutionary novelty can arise as information transcoding from the genotype, the level where the model-building blocks are specified, to the phenotype, where selection takes place. **Constructive novelty**: Evolutionary novelty can arise as a side effect of selection on a tangentially related trait. In the case of an evolutionary transition, this can lead to a new level of organisation. The figure sketches two examples of models that capture these forms of novelty.

In models where there is an explicit selection on a particular phenotype, the lower (molecular) and higher (phenotype + fitness) levels are both explicitly defined, so adaptation that occurs at the higher level is not novelty. Instead, the kind of novelty shown by these models lies in the mechanism that evolves to generate the traits under selection, like the morphogenetic processes and gene expression dynamics. These novel mechanisms effectively generate one or more levels of information transcoding between the genotype and the predefined target phenotype. We call this ‘between-level novelty’ (Figure 1). These models show how the GP map becomes functionally more complex over evolutionary time, resulting in a variety of different possible developmental mechanisms. Once a specific developmental mechanism has evolved, it is interesting to characterise the outcome, e.g. in terms of gene regulatory network architecture, or in terms of its local fitness landscape. We can thus treat the model outcome as a non-novelty model.

### Constructive novelty

Alternatively, models can show the evolution of novelty as a side effect of selection on an unrelated trait, rather than as a way to organise the information flow from genotype to fitness. In these models, the evolved phenotype is not preconceived, but arises through the emergent organisation of the model’s building blocks. The result can be the construction of a novel level of organisation: a major evolutionary transition [1]. To emphasise that evolution is a constructive process [27], we call this constructive novelty (Figure 1). However, models that show constructive novelty extend beyond hierarchical evolutionary transitions. We propose that a theory of construction can include any trait/property that opened up and structured a new space of possible evolutionary innovations. We will give examples of models that display these two types of novelty in the context of development, and discuss what they reveal about how novelty arises.

## Between-level novelty: modelling the evolution of developmental pattern formation

One of the most thoroughly studied processes in animal development is the formation of segments, repeated elements along the main body axis of bilateral animals. In particular annelid worms [28], arthropods [29] and chordates [30] are clearly segmented, with a remarkable variety of different mechanisms to generate them during embryonic development; this has sparked a heated debate on the origins of a segmented body plan [31–36]. Multiple evo–devo models were constructed to understand the evolution of the developmental mechanisms that generate segments (reviewed in [37]). While segmentation itself can be considered an evolutionary novelty, in these models a segmented (striped) gene expression is explicitly selected for, and is therefore a trait that is assumed to be possible or present – not novelty. Instead, these models can reveal the space of possible novel, between-level developmental mechanisms that can evolve.

Multiple novel mechanisms evolved in each of these models, highlighting the generative potential of between-level evolution. Broadly categorised, segments can be generated simultaneously or one by one sequentially. Examples of simultaneous mechanisms that evolved are: hierarchical mechanisms (reminiscent of the mechanism in Drosophila) [38–40], reaction-diffusion mechanisms [38,41], and noise-amplifying mechanisms [42]. Sequential mechanisms include: stereotyped asymmetric divisions in a growth zone (reminiscent of annelid worms) [42]; timed, segment-specific determination of a band [43]; and the transformation of gene expression oscillations into a spatial pattern (like the clock-and-wavefront mechanism in vertebrates) [39,41–44].

Whether simultaneous or sequential segmentation evolved, depended for a large part on the predetermined morphogen and growth dynamics in the tissue that scaffolded the evolution of the novel mechanism. Simultaneous segmentation dominated if the tissue to be patterned was static – no cell divisions and a nonmoving morphogen pattern, while moving morphogen gradients (either due to decay and/or tissue growth) resulted more often in sequential mechanisms. The evolved segmentation mechanism could itself function as a scaffold for the evolution of additional developmental processes, such as the cessation of axial growth [42] or regional body axis differentiation [43]. Thus, we can already observe in models the progressive co-option of evolved mechanisms into newly evolving mechanisms, suggesting that this is an iterative process.

While the presence of developmental scaffolds is historically contingent for any particular species, it is an open question whether there can be long-term selection on ‘good scaffolds’ that facilitate continual novelty. Scaffolds could be developmental signalling pathways (e.g. Wnt, Auxin signalling), developmental dynamics (e.g. oscillations) or even entire physical structures (e.g. segments) – but it is still unclear what properties makes for a ‘good scaffold.’ Observing whether this type of selection occurs and which type of scaffold is selected, will require models that are able to simulate multiple events of evolutionary novelty, either at the in-between level or constructively.

## Constructive novelty: models of the evolution of multicellularity

Models of evolutionary transitions of individuality [1,45] can provide examples of constructive novelty, as the higher-level individual is itself novel, and serves as a novel context in which new traits and functions can arise. A model can capture this type of novelty when higher-level individuality arises from the interactions of lower-level individuals without being explicitly modelled or selected for. When it comes to evolution of development, the relevant transition is the evolution of multicellularity, when the first form of development arose.

Models have worked on different aspects of this transition. Emerging multicellular life cycles can be viewed as between-level novelty when they arise as a side effect of explicit selection on the multicellular group and cells’ gene expression patterns [46]. A constructive form of novelty can instead be identified in a spatially structured model of cells living in a toxic environment [47]. These cells could evolve the switching probability between degrading the toxin and dividing. Different populations rapidly evolved various multicellular strategies for protecting a group of reproductive cells by surrounding them with differentiated, toxin-degrading cells. The spatial arrangement of these multicellular structures, and the proto-developmental dynamics that generated it are a form of constructive novelty, as they constitute a level of organisation higher than that where the modelled fitness operates.

In our recent work [48], we modelled a population of cells searching for resources by chemotaxis. In response to this environment, cells evolved to adhere to each other, since groups of cells performed emergent collective chemotaxis. Adhesion itself was not novel, as it was explicitly included in the model. Instead, the selection pressure to adhere to other cells does constitute constructive novelty: the model rewarded exclusively individual cells and therefore the selection for adhesion arose as a side effect of individual cells’ competition (and indeed unicellularity evolved when the chemotactic signal was too unstable for cells to collectively move towards it). In follow-up work, we let cells coevolve adhesion with the regulation of their behaviour. As adhesion evolved, cells found themselves progressively more in a biotic environment – the cell cluster – and adapted to it. Comparing cells that evolved with or without cell adhesion – the latter representing the unicellular ancestor – we found drastically different strategies regulating when cells migrated towards resources and when they divided [49]. This showed that the same behaviour can be exploited in different ways in the transition to multicellularity. The regulatory dynamics of cells in adhesive clusters gave rise to emergent morphogenetic expansion and extension of the cluster as it migrated towards resources, suggesting a path towards development as constructive novelty in this model.

We note that models showing constructive novelty tend to capture ‘messy’ solutions, in which the different levels of organisation are not clearly separated. This reflects the situation of many extant species that have evolved a host of alternative colonial solutions [50]. These solutions challenge modelling approaches that predetermine the social role of different cell types (e.g. cooperation, policing, defectors, etc.) or the aggregation strategy (e.g. staying together vs. coming together).

## Between-level and constructive novelty: models of genome evolution

An important aspect of multicellular development is the functional specialisation of different cells, also known as division of labour. From an evolutionary view point, division of labour can be beneficial to the group when specialised cells are more efficient than generalists. Models of the evolution of division of labour often focus on identifying conditions where the benefit of cell specialisation for the group outweighs a metabolic or informational cost paid either by individual cells or by the group [51–56]. In these models, the genomic basis of phenotypes has been abstracted away, and the space of possible phenotypes and their functional relation have been predetermined, often by means of a trade-off. This setup does not allow for evolutionary novelty.

Instead, a possible avenue for modelling novelty in the context of division of labour could be to investigate the evolution of the trade-off itself (as was done for evolution of microbial cross-feeding [57]), or the emergence of differentiation as a side effect of intracellular dynamics [58]. A third approach, which is a form of between-level novelty, is to study how genomes evolve to encode information for organising division of labour. This could happen through genomic structuring of gene regulation, or, as we consider here, by biasing the effect of mutations to generate useful mutant phenotypes [23,59]. We used this latter perspective to understand the development of the multicellular colonies of *Streptomyces coelicolor*, after it had been found that specialised antibiotic-producing cells within the colony are generated through mutations rather than gene expression regulation [60].

We constructed a spatial model of *Streptomyces* genome evolution and division of labour [61]. The rates of growth and antibiotic production of each cell in a colony-depended on the gene content (growth genes or antibiotic genes) of their linear genome, with an explicit trade-off: more growth genes resulted in higher replication rates but lower antibiotic production. The genome could also host one or more mutational hotspots, i.e. genomic sites where genome deletion is likely. In this model, competition for space between colonies resulted in the evolution of large, clearly structured genomes with many antibiotic genes and growth genes, separated by a cluster of mutational hotspots. Wild-type cells possessing such genomes rapidly divided to expand the colony, but were unable to produce antibiotics due to the trade-off. However, a sizeable fraction of their offspring was mutants due to a hotspot activation, which resulted in genomes containing many antibiotic genes but where all growth genes were deleted. These sterile mutants therefore produced many antibiotics that protected the colony.

Novelty in this model is not in the evolution of division of labour – we selected for that with the explicit trade-off. Evolutionary novelty in this model is twofold: (1) constructive novelty, in the form of the emergence of between-colony competition driving the evolution of genome structuring (a type of downward causation [62]), and (2) between-level novelty in the form of genome structuring, resulting in nonrandom mutational dynamics. This modelling approach could be extended to explain the widespread usage of DNA manipulation for cell differentiation in eukaryotic multicellularity [63–65].

## Constructive novelty: models of the evolution of morphogenesis

During morphogenesis, cells interact with one another mechanically and chemically, generating complex patterns of cell differentiation, migration, death, and division. This process gives rise to the functional form of the adult organism. While morphogenetic processes are required for generating adaptive and in many cases essential phenotypes, it is unlikely that a morphogenetic process evolved in the first instance because it is adaptive, for the following reasons. First, the current developmental program has to have new morphogenetic processes in its close mutational neighbourhood, so that a few mutations can bring about a particular phenotype. So some genotypes may be better suited than others to evolve certain phenotypes, and thereby generate the potential for novelty (this is related to the scaffolding discussed in the section on segmentation) [66]. Second, novel structures are rarely adaptive in isolation; they must be used in a novel way that is meaningful in the current environment. The evolution of morphology should be accompanied by the evolution of the behaviour that makes it functional in its ecological context (provided that such context exists [67]). Behaviour may be an orthogonal phenotypic element, e.g. if it is under control of independent genetic factors, which could be unlikely to coevolve immediately with the emergence of the new morphological feature itself. Thus, novel structures may require the synergy of multiple traits evolved in parallel or sequentially [68]. We therefore expect novel morphogenetic processes to arise as a side effect of another evolutionary process.

We see three possible modelling approaches: (1) making a model in which morphogenesis results from selecting a target morphology, (2) making a model where morphology is selected for by the function the structure has in the interaction between the organisms and the environment, (3) select for tangential traits and let morphogenesis evolve as a side effect. While (1) and (2) would model between-level novelty (but have not been tried), (3) can show constructive novelty, and has been studied in a model by Hogeweg about 20 years ago [69,70].

In this model, individuals were selected based on the number of different cell types generated by their evolved gene regulatory network, which governed gene expression and cell adhesion, leading to cell migration, division, and death in a developing tissue. While the fittest individuals – those with the largest number of different cell types – typically formed more or less round tissues, their close mutants formed a large diversity of striking shapes, like hollow balls, elongated stems, buds, and branches. These shapes were generated by morphogenetic processes that resembled those in living organisms, such as meristematic growth and differentiation, engulfing, intercalation and elongation, and budding, which themselves resulted from the evolved coupling between cell differentiation, adhesion, migration, division, and death [69].

The additional cell movement due to morphogenesis could even reduce the number of cell types, leading to lower fitness of the mutants. Nevertheless, new shapes appeared throughout evolution as side branches of the line of descent, while morphologically similar mutants could be separated by many generations [70]. The evolution of morphogenesis is a form of novelty in this model. As part of the mechanism that induces cell differentiation, morphogenesis is between-level novelty. However, morphogenesis is also a consequence (a side effect) of a tissue with multiple cells types, making it constructive novelty. Once shape has evolved, changes in behaviour could subsequently functionalise the new feature in a process of exaptation.

## Discussion

In the models presented here, pattern formation and morphology served as proxy for fitness. In reality, patterns and shapes are ultimately selected on the basis of the function (metabolic, motoric, sensory, etc.) they support. How can we integrate the function of the resulting phenotypes into evolutionary models?

We explored one way in which function arises as constructive novelty in our work on the evolution of multicellularity. There, the function of collective chemotaxis enabled the cluster to find resources and physically displace unicellular populations, but was not directly under selection. Function can also be selected for explicitly, so that the mechanisms and morphological structures to perform such a function emerge as between-level novelty. This approach is regularly taken in the field of Artificial Life, where studies explore selection for functions such as reaching for a resource or performing locomotion using articulated limbs [71], or soft tissue [72], often with surprising results [73]. The building blocks in these studies do not correspond well to biology, but we believe that there is much insight that can be gained by integrating this approach within a biological model of evolution of development.

A second challenge becomes how to analyse the results of these models, as these models will show progressively more open-endedness. One possibility is to exploit their historical development, i.e. to use the previous, simpler models as null hypotheses. This approach has recently been used to show that some quantitative population genetics models of phenotypic evolution perform poorly near large discontinuities of the genotype–phenotype map [74]. A second possibility is to exploit bioinformatic and statistical tools developed for the analysis of ‘real’ genomes, such as multiple sequence alignments and phylogenetic trees (and conversely, the validity of these tools can be tested with evolutionary models [75]). Third, developmental mechanisms evolved in these models can be compared with known mechanisms, similarly to how models of development are compared with experiments [10,12]. Evolved mechanisms that do not have such ‘real-life’ resemblance can be considered model predictions, in that they may still be identified in as yet unknown organisms.

We have shown here several examples of models in which specific novel traits emerge. How can we extract a general understanding of the evolution of novelty? We (and others [76]) expect that, as more of such models are constructed, coarse-grained similarities between models will appear, pointing to broader principles of novelty evolution. One example is that evolution structures the GP map, so that mutants acquire useful functions. We showed an example of this in the model of *Streptomyces* development. This structuring of the GP map was also observed in a model of RNA evolution [59] and in a model of microbial adaptation to fluctuating environments [23], despite the fact that the building blocks of these models were completely different. While some models will share some of their evolved mechanisms, others may serve as counter-examples (perhaps by evolving the same feature through a different mechanism), limit cases, etc. Counterexamples are common in Biology, and we expect that models of the evolution of novelty will be no different in this respect. If this expectation holds, then no single unifying definition of novelty may be possible. In that case, a web (or a rhizome [77]) of models captures best the evolution of novelty as it occurs in ‘real’ biological systems. Evolution continually generates new possibilities, also in the mechanisms of evolution itself, which is reflected in the complexity and diversity of life.

## Summary

- The generation of novelty is the most striking outcome of evolution.
- Computational evo–devo models can capture some aspects of evolutionary novelty.
- Novelty is captured in one of two ways: as ‘between-level novelty’ or as ‘constructive novelty.’
- Between-level novelty arises as functional complexification of the developmental Genotype–Phenotype Map, between the level at which the model is specified and the level of selection.
- Constructive novelty results in the emergence of a novel level of organisation tangential to or above the level of selection.

## Open Access

Open access for the present article was enabled by the participation of University of Cambridge in an all-inclusive *Read & Publish* agreement with Portland Press and the Biochemical Society under a transformative agreement with JISC.

## Abbreviations

GP: genotype-to-phenotype
